# Influence of a Brief Episode of Anesthesia during the Induction of Experimental Brain Trauma on Secondary Brain Damage and Inflammation

**DOI:** 10.1371/journal.pone.0019948

**Published:** 2011-05-19

**Authors:** Clara Luh, Katharina Gierth, Ralph Timaru-Kast, Kristin Engelhard, Christian Werner, Serge C. Thal

**Affiliations:** 1 Department of Anesthesiology, Medical Center of the Johannes Gutenberg-University, Mainz, Germany; 2 Institute of Physiology and Pathophysiology, Medical Center of the Johannes Gutenberg-University, Mainz, Germany; University of Muenster, Germany

## Abstract

It is unclear whether a single, brief, 15-minute episode of background anesthesia already modulates delayed secondary processes after experimental brain injury. Therefore, this study was designed to characterize three anesthesia protocols for their effect on molecular and histological study endpoints. Mice were randomly separated into groups that received sevoflurane (sevo), isoflurane (iso) or an intraperitoneal anesthetic combination (midazolam, fentanyl and medetomidine; comb) prior to traumatic brain injury (controlled cortical impact, CCI; 8 m/s, 1 mm impact depth, 3 mm diameter). Twenty-four hours after insult, histological brain damage, neurological function (via neurological severity score), cerebral inflammation (via real-time RT-PCR for IL6, COX-2, iNOS) and microglia (via immunohistochemical staining for Iba1) were determined. Fifteen minutes after CCI, the brain contusion volume did not differ between the anesthetic regimens (sevo = 17.9±5.5 mm^3^; iso = 20.5±3.7 mm^3^; comb = 19.5±4.6 mm^3^). Within 24 hours after injury, lesion size increased in all groups (sevo = 45.3±9.0 mm^3^; iso = 31.5±4.0 mm^3^; comb = 44.2±6.2 mm^3^). Sevo and comb anesthesia resulted in a significantly larger contusion compared to iso, which was in line with the significantly better neurological function with iso (sevo = 4.6±1.3 pts.; iso = 3.9±0.8 pts.; comb = 5.1±1.6 pts.). The expression of inflammatory marker genes was not significantly different at 15 minutes and 24 hours after CCI. In contrast, significantly more Iba1-positive cells were present in the pericontusional region after sevo compared to comb anesthesia (sevo = 181±48/mm^3^; iso = 150±36/mm^3^; comb = 113±40/mm^3^). A brief episode of anesthesia, which is sufficient for surgical preparations of mice for procedures such as delivering traumatic brain injury, already has a significant impact on the extent of secondary brain damage.

## Introduction

Traumatic brain injury (TBI) triggers a cascade of secondary events that ultimately lead to a delayed and progressive destruction of surrounding brain tissue, and the pathophysiological processes of this secondary expansion are poorly understood. To examine this essential phenomenon in more detail, standardized in vivo models are necessary. In mice, controlled cortical impact (CCI) is one of the most commonly applied experimental TBI models. The CCI model is ideal to induce very reproducible primary lesions but requires background anesthesia during the surgical preparation. However, anesthetics alter brain metabolism, cerebral blood flow, and physiological variables like arterial blood gases, mean arterial blood pressure, or cardiac output [1]. Likewise, anesthetics have neuroprotective effects when administered prior to cerebral ischemia for several hours after insult [2], [3]. Anesthesia itself may therefore already influence secondary events like cerebral perfusion, excitotoxicity, inflammation, and apoptosis [4], which will lead to changes to the extent of neuronal injury and functional outcome as demonstrated by one hour of anesthesia with different protocols following rat TBI [5].

The impact of very brief episodes of background anesthesia on secondary pathophysiological mechanisms has so far not been determined. Therefore, the present study aims to investigate the influence of sedation for 15 minutes with three common sedation protocols (isoflurane [iso], sevoflurane [sevo] and an intraperitoneal [i.p.] combination anesthesia [comb]) on histopathological damage, neurological function, and cerebral inflammation in a traumatic brain injury model in mice.

## Results

### Mortality and physiological parameters

All animals (male C57Bl/6N mice) survived the 15-minute or 24-hour observation period. Physiological parameters (15 minutes after CCI) were within the normal range and were not different between the experimental groups (**Table 1**) with the exception of comb animals, which developed a reduced pH (7.21±0.1) and demonstrated an elevated paCO_2_ (57.0±4.7 mmHg, p<0.05 vs. iso or sevo). The systolic blood pressure with comb anesthesia was significantly higher (143±33 mmHg) compared to sevo (100±22 mmHg) five minutes after CCI, but not different between sevo and iso.

**Table 1:** Physiological parameter.

	sevo	iso	comb
arterial pH (15 min after CCI)	7.25±0.3	7.29±0.1	7.21±0.1
paO_2_ [mmHg] (15 min after CCI)	211±12	238±19	131±46
paCO_2_ [mmHg] (15 min after CCI)	39.7±6.8	35.5±3.7	$ ^,^ *57.0±4.7
Syst. BP [mmHg] (5 min before CCI)	103±24	110±13	136±29
Syst. BP [mmHg] (5 min after CCI)	100±22	107±20	*143±33
Rect. Temp. [°C] (5 min after CCI)	36.1±0.8	36.2±0.8	36.0±0.7

paO_2_/paCO_2_ = arterial O_2_/CO_2_ partial pressure determined 15 minutes after trauma; Syst. BP = non-invasively determined systolic blood pressure; Rect. Temp = rectal body temperature;

*p<0.05 vs. sevo,

$ p<0.05 vs. iso.

### Influence of the anesthetic regimen on histological brain damage

**Figure 1:**
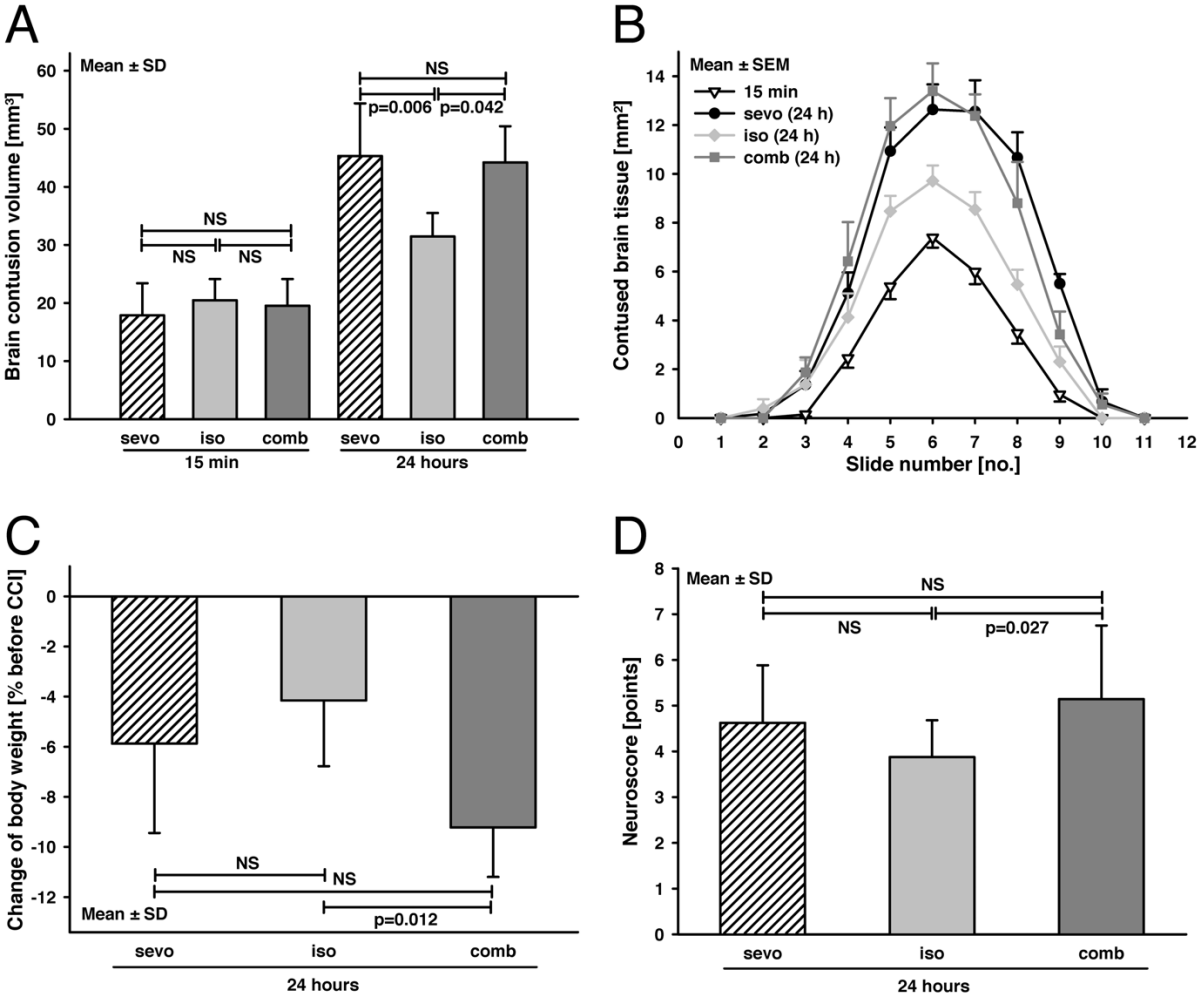
Histological brain damage and neurological function. (**A**) The contusion volume was determined 15 minutes and 24 hours after experimental brain trauma (CCI). The primary lesion 15 minutes after CCI was not significantly different between the anesthetic regimens. Within 24 hours, brain contusions increased significantly in all groups. Sevo-anesthetized animals experienced a significantly larger contusion volume compared to the isoflurane and comb groups (group size: 15 minutes, n = 6 per group; 24 hours, n = 8). (**B**) The contusion areas of all groups obtained from 11 coronal brain sections (750-µm interval) cut from rostral (no. 1) to dorsal (no. 11) 15 minutes and 24 hours after CCI. The graph illustrates that the secondary expansion of brain damage occurred in all brain areas (mean ± S.E.M.). (**C**) The body weight was determined 24 hours after CCI and was compared to the value obtained before trauma as the general marker for the well-being of the animals. Animals anesthetized with comb lost significantly more body weight compared to iso. The other anesthetic regimens were not significantly different. (**D**) Neurological function was determined by the neurological severity score (NSS; 0 point = no impairment; 10 points = maximal impairment, (**Table 2**) 23 hours after CCI. NNS scores of comb were significantly worse compared to iso. The other groups were not significantly different. (sevo = sevoflurane; iso = isoflurane; comb = i.p. injection of midazolam, fentanyl and medetomidine; data are presented as mean ± S.D.; NS = not significant; p-values are adjusted for multiple comparison by Bonferroni).

The primary lesion at 15 minutes after trauma was similar in all groups (sevo = 17.88±5.53 mm^3^; iso = 20.47±3.67 mm^3^; comb = 19.53±4.6 mm^3^; **Figure 1 A**). Brain contusion volume increased at 24 hours from CCI in all groups (sevo = 45.32±9.04 mm^3^; iso = 31.46±4.04 mm^3^; comb = 42.11±8.27 mm^3^; p = 0.006 vs. 15 min). Animals anesthetized by sevo (p = 0.006) or comb (p = 0.042) developed a significantly larger contusion volume compared to iso after 24 hours.

**Table 2:** Criteria of the Neurological Severity Score (NSS).

Task	Points
Presence of mono- or hemiparesis	1
Inability to walk on a 3-cm-wide beam	1
Inability to walk on a 2-cm-wide beam	1
Inability to walk on a 1-cm-wide beam	1
Inability to balance on a 1-cm-wide beam	1
Inability to balance on a round stick (0.5 cm diameter)	1
Failure to exit a 30-cm-diameter circle (for 2 min)	1
Inability to walk straight line	1
Loss of startle behavior	1
Loss of seeking behavior	1
Maximum total	10

One point is awarded for failure to perform a task.

### Influence of the anesthesia on functional outcome

Body weight was measured prior to surgery and 24 hours after CCI as a general marker for well-being and was compared to values obtained prior to trauma (sevo = −5.9±3.6%; iso = −4.2±2.6%; comb = −9.2±2%; **Figure 1 C**). Loss of body weight was significantly higher in comb compared to isoflurane-anesthetized animals (p = 0.012).

All animals demonstrated an impaired neurological function at 23 hours after trauma (sevo = 4.6±1.3 points; iso = 3.9±0.8 points; comb = 5.5±1.8 points). Neurological function was significantly worse with comb compared to iso (p = 0.027) but not to sevo (**Figure 1 D**).

### Inflammatory marker gene expression after brain trauma

**Figure 2:**
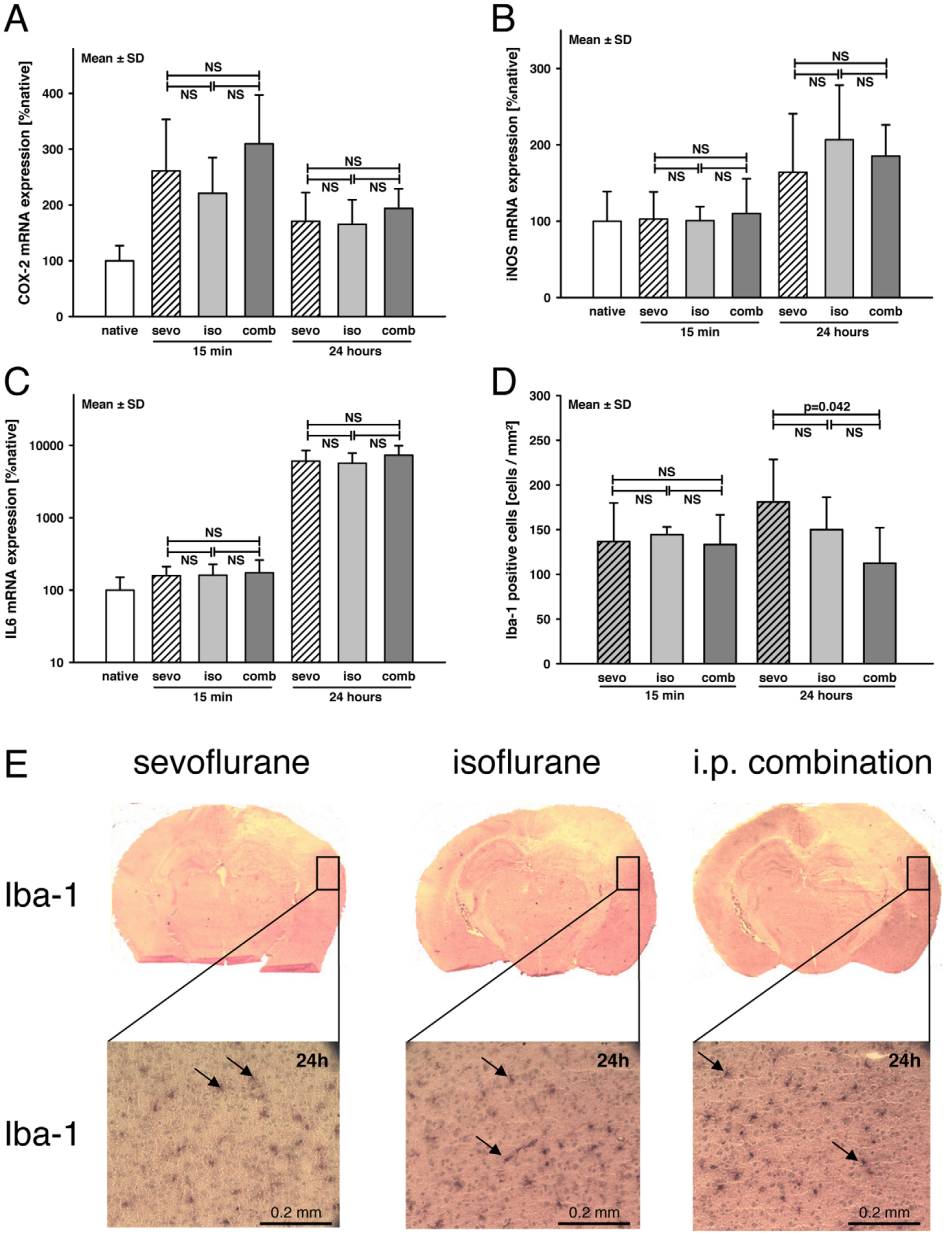
Expression of inflammatory marker genes and microglia activation. **A–D:** The mRNA expression was determined in contused brain tissue. Expression of the inflammatory marker genes COX-2 (**A**), iNOS (**B**), and IL6 (**C**) was not significantly different between the anesthetic protocols at 15 minutes and 24 hours after experimental brain trauma (group size: naïve, n = 9; 15 minutes, n = 6; 24 hours, n = 8; data are presented as mean ± S.D.). **D:** As a marker for activated microglia, immunohistochemical analysis of Iba-1-positive cells was performed in the pericontusional tissue that revealed a significantly increased activation in sevo animals vs. comb (n = 8 per group; sevo = sevoflurane; iso = isoflurane; comb = i.p. injection of midazolam, fentanyl and medetomidine; native = no surgery; NS = not significant; p-values were adjusted for multiple comparison by Bonferroni). **E:** Example pictures of the Iba-1-stained slides of animals 24 hours post-injury (20× magnification). The number of activated microglia was determined at the border zone adjacent to the damaged brain tissue. Iba-1-positive cells are labeled dark brown (arrows).

Expression of the inflammatory marker genes COX-2 (**Figure 2 A**), iNOS (**Figure 2 B**), and IL6 (**Figure 2 C**) was not significantly different between the anesthetic protocols at 15 minutes and 24 hours after experimental brain trauma.

### Iba-1 activity

Immunoreactive glial cells were quantified as a marker for cerebral inflammation by staining for Iba-1. Fifteen minutes after CCI, all animals presented low level inflammation (128±42 Iba-1-positive cells/mm^3^; **Figure 2 D**) independent of the group. Twenty-four hours after trauma, the number of Iba-1-positive cells in the pericontusional region increased in the sevo group (sevo = 181±48/mm^3^; iso = 150±36/mm^3^; comb = 113±40/mm^3^).The number of Iba-1-positive cells was significantly higher in the sevo group compared to the comb group (p = 0.042).

## Discussion

This study demonstrates that even brief episodes of background anesthesia already influence secondary damage following experimental brain injury. The key findings of the study are the following: 1) sevoflurane (sevo) and the combination i.p. anesthesia (comb) increase the extent of brain damage at 24 hours from CCI when compared to isoflurane (iso), and, accordingly, neurological function with comb anesthesia was significantly worse compared to isoflurane anesthesia; 2) microglial activation was significantly higher after sevoflurane anesthesia compared to comb; and 3) despite differences in the extent of parenchymal damage and microglia activation, local inflammatory marker gene mRNA expression did not differ between the anesthesia protocols.

To investigate traumatic brain injury, multiple models have been developed in rodents, ranging from the induction of heat or cold lesions on the brain surface or the compression of brain parenchyma with a piston or water cushion [6], [7]. One of the most frequently applied models is the controlled cortical impact (CCI), which utilizes a piston to penetrate the brain at a defined velocity, depth and impact duration. The surgery takes 10 to 15 minutes to deliver a CCI in mice and is performed under background anesthesia, which is then discontinued within 5 minutes after the trauma is delivered. However, anesthesia per se may confound data due to its effects on secondary mechanisms. In traumatic brain injury research, the ideal anesthetic agent should fulfill the following criteria: 1) no influence on brain lesion extent, 2) no influence on neurological function, and 3) no influence on secondary mechanisms such as inflammation. The study, therefore, evaluated the impact of current anesthetic protocols on key experimental endpoints after CCI by comparing the typical anesthesia protocols that are applied during experimental procedures. Isoflurane and sevoflurane currently replace halothane in laboratory practice and were therefore selected as examples of inhalative agents. Both substances share the advantage of fast sedation induction and termination speed but demonstrate neuroprotective potency [8]. The protective effects are attributed to the upregulation of nitric oxide synthase with increased cerebral perfusion and mitochondrial ATP-regulated potassium channel activity and reduced excitotoxic and metabolic stress and apoptotic cell death [8]. In recent studies, intraperitoneally injectable anesthetics have become a popular alternative to produce surgical anesthesia with limited vascular effects. The combination of the anesthetics fentanyl, midazolam, and medetomidine was selected as an example used in spontaneously breathing animals [9], [10], [11], [12]. The substances can be antagonized specifically by naloxon, flumazenil, and atipamezol to promptly terminate sedation within 1–3 minutes and demonstrate little effect on cerebral perfusion and intracranial pressure [13], [14]. The downside of the protocol is the use of six drugs with different pharmacological properties. The opposing effects of the anesthetic agents of the comb protocol impede a reliable estimation on the impact of secondary brain damage following CCI. To date, no published information is available on the differential effect of the comb treatment on secondary brain damage and the underlying mechanisms.

To determine the influence of the sedation protocols on secondary lesion expansion, primary initial lesion volume was determined at 15 minutes after trauma. The results demonstrate that the primary contusion is independent of the anesthetic agent. Over time, the brain contusion expands into surrounding healthy brain tissue. In contrast to the primary lesion, this expansion differs between individual anesthesia protocols. Isoflurane anesthesia resulted in the lowest histological damage, while sevoflurane and comb anesthesia caused greater damage. Accordingly, animals anesthetized with isoflurane showed the best neurological function and the lowest body weight loss. While a recent study has shown that the application of seven different anesthetic agents for one hour following traumatic brain injury influenced the histopathological damage [5], the present study demonstrates for the first time that even very brief episodes of anesthesia (10–15 minutes) already influence secondary brain damage.

Mechanisms of anesthesia-induced alterations of secondary brain damage are not fully understood. The present findings are in accordance with a recent study, in which isoflurane compared to sevoflurane given for 30 minutes after trauma reduced lipid peroxidation more efficiently [15], which plays a crucial role in secondary brain damage [16]. The beneficial action of isoflurane is likely to be multifactorial and may be attributed, in part, to cerebral vasodilation and attenuation of excitotoxicity. Isoflurane reduces glutamate release [17], blocks NMDA receptors more effectively than sevoflurane [18], and reduces NMDA-mediated calcium influx [19]. Additionally, isoflurane increases cerebral blood flow [20]. In contrast, sevoflurane-treated animals demonstrate higher intracranial pressure (ICP) (and lower cerebral perfusion pressure [CPP] levels) compared to isoflurane after head trauma [21], which may contribute to the observed differences. An increase in ICP and a reduction in CBF is known to contribute to secondary contusion expansion after head trauma [22].

Cerebral inflammation is implicated as one of the key parameters for secondary lesion expansion. The inflammatory response is characterized by the early production of pro- and anti-inflammatory cytokines [23], the upregulation of inducible nitric oxide synthase [24], and the accumulation of neutrophils in the injured brain [25]. Despite differences at the histological level, the expression of inflammatory marker genes did not differ between the study groups. In contrast, the microglia were significantly more activated after the sevo compared to the comb protocols. Although microglia-mediated inflammation seems to be affected by the anesthesia, similarities in the inflammatory mRNA expression profile suggest a lack of a direct aggravating influence of anesthetics on cerebral inflammation. A possible alternate explanation for the observed differences in histological and neurofunctional outcome is “preconditioning” by volatile anesthetics. In experimental stroke, exposure with 1.1 or 2.2% isoflurane prior to ischemic stroke prevented apoptosis [26]. Exposure to volatile anesthetics 5 minutes prior to CCI may already be sufficient to alter or “precondition” tissue to become less sensitive to mechanical injury.

Physiological distinctions between the groups should also be considered as possible explanations for the differences in histological and neurofunctional outcomes. In this study, physiological variables were not different between isoflurane- and sevoflurane-anesthetized animals. Therefore, physiological variables alone cannot explain the difference in histological brain damage and neurological function between sevoflurane- and isoflurane-anesthetized animals. In contrast, differences in the arterial blood gases and systolic blood pressure were observed between volatile and comb anesthesia protocols. Increased paCO_2_ level in the comb group may result in increased cerebral blood volume and intracranial pressure and may have contributed to the large histological brain damage compared to isoflurane. Additionally, systolic blood pressure was significantly higher in comb animals. Improved blood pressure did not result in a smaller contusion volume, although an increase in blood pressure may improve collateral cerebral blood flow in injured regions [27]. Alternatively, it could also be argued that a higher systolic blood pressure may have enhanced edema formation due to bleeding into the traumatized brain region [28], [29].

### Conclusion

Selection of an appropriate anesthetic agent seems to be essential, as initial anesthesia influences delayed secondary lesion expansion probably because of the modulation of neuronal cell death. Importantly, cerebral inflammation seems to be independent of the selected anesthetic agent. Based on this study, sevoflurane anesthesia may be superior to induce surgical anesthesia with normal physiological parameters along with substantial lesions to determine histological and neurological outcome.

## Materials and Methods

A total of 51 male C57Bl/6N mice that weighed between 18 and 24 g and were approximately 2 to 3 months of age (Charles River Laboratory, Sulzfeld, Germany) were investigated. Prior to and during experiments, the animals were cared for in compliance with institutional guidelines of the Johannes Gutenberg-University, Mainz. The Animal Ethics Committee of the Landesuntersuchungsamt Rheinland-Pfalz gave approval for all the experiments (protocol number: G08-1-012).

### Traumatic brain injury

Animals were randomly separated into three different anesthetic regimens: those that received isoflurane (iso), sevoflurane (sevo), or a combination (comb) of midazolam (5 mg/kg; Ratiopharm, Ulm, Germany), fentanyl (0.05 mg/kg; CuraMed, Karlsruhe, Germany), and medetomidine (0.5 mg/kg; Pfizer, Karlsruhe, Germany). To ensure the same trauma severity in all animals, survival duration was revealed after the induction of trauma (15 minutes = technical control or 24 hours). The comb protocol was terminated by subcutaneous (s.c.) injection of flumazenil (0.5 mg/kg; Hoffmann-La-Roche, Grenzach-Wyhlen, Germany), naloxon (1.2 mg/kg; Inresa, Freiburg, Germany), and atipamezol (2.5 mg/kg; Pfizer, Karlsruhe, Germany). Anesthesia was induced in a bell jar filled with 4% iso or 6% sevo and maintained via facemask (1.4% iso or 3.5% sevo in 40% O_2_ and 60% N_2_). In animals randomized into the comb group, anesthetics were injected intraperitoneally, and an air mixture (40% O_2_ and 60% N_2_) was supplied via facemask in spontaneously breathing animals. The selected dosage was the minimal amount required to inhibit movement of the mice during the surgical procedure and was determined in a pilot study (data not shown).

The brain trauma model was performed as previously described [30]. Animals were placed in a stereotactic frame (Kopf Instruments, Tujunga, USA) and were subjected to brain trauma by CCI. A craniotomy (4×4 mm) was performed using a high-speed drill over the right parietal cortex between the sagittal, lambdoid, and coronal sutures. A custom-fabricated pneumatically controlled cortical impactor (L. Kopacz, Mainz, Germany) was placed directly onto the cortical surface of the brain. For all animals, an impactor tip was used that had a diameter of 3 mm, an impact velocity of 8 m/s, an impact duration of 150 ms and a brain penetration depth of 1 mm. Immediately after cortical impact, the craniectomy was closed with the initially removed bone and fixed with conventional tissue glue (Histoacryl, Braun-Melsungen, Melsungen, Germany). The wounds were closed with filament sutures at the end of the surgery.

Rectal temperature was maintained constant at 37°C by a feedback-controlled heating pad (Hugo Sachs, March-Hugstetten, Germany) and recorded 5 minutes before and 5 minutes after CCI. At the end of the preparation, anesthesia was discontinued. Animals that survived 15 minutes post-CCI remained on the heating pad until their brains were removed. Mice surviving CCI for 24 hours were placed in their individual cages and allowed to recover for 6 hours in an incubator heated to 33°C and at a humidity of 35% (IC8000, Draeger, Germany).

### Experimental groups

Animals were randomly assigned to six trauma groups and a native (no surgery) control group. The anesthesia was initiated prior to surgical preparation and was terminated immediately after CCI and wound closure to ensure a similar duration of anesthesia:

Group 1: iso +15 min survival (n = 6)
Group 2: sevo +15 min survival (n = 6)
Group 3: comb +15 min survival (n = 6)
Group 4: iso +24 hr survival (n = 8)
Group 5: sevo +24 hr survival (n = 8)
Group 6: comb +24 hr survival (n = 8)
Group 7: native animals (n = 9, 3 animals per anesthetic regimen)

### Measurement of physiological parameters

Blood pressure was measured 5 minutes before and 5 minutes after CCI at the tail using a modified non-invasive blood pressure system (RTBP 2000, Kent, USA) as previously described [12]. Cuff pressure signals were recorded with a sample rate of 100 Hz (A/D converter: PCI 9112, Adlink Technology, Taiwan; PC software: Dasylab 5.0, measX, Germany) and analyzed offline (Flexpro 6.0, Weisang, Germany) for systolic blood pressure. In animals that survived 15 minutes, arterial blood samples were drawn from the common carotid artery. Arterial blood gas concentrations and blood glucose levels were determined with the blood gas analyzer ABL800 BASIC (Radiometer Medical ApS, Brønshøj, Denmark).

### Histological evaluation

At the end of the observation period, the animals were euthanized in deep anesthesia by repeating the initial anesthetic regimen or in the native group by immediate cervical dislocation after allocation to one of the three anesthesia regimens.

The brains were carefully removed, frozen in powdered dry ice, and stored at −20°C. Brains were cut in the coronal plane with a cryostat (HM 560 Cryo-Star, Thermo Fisher Scientific, Walldorf, Germany). The first slide was defined according to the Mouse Brain Library atlas as bregma +3.14 mm (www.mbl.org). Ten-micron-thick sections were collected every 750 µm, placed on Superfrost plus slides (Thermo Fisher Scientific, Germany) and stained with cresyl violet. For mRNA quantification, tissue samples of the right upper quadrant were collected by separation of the remaining tissue between two intervals in the region with contused brain tissue and were stored at −80°C. The area of both hemispheres and contused brain tissue were determined with a computerized image system (Optimas 6.51, Optimas Corporation, Bothell, USA) by an investigator blinded to the group allocation. The contusion volume and hemispheric brain volume were calculated based on the contusion and brain areas obtained from 11 consecutive sections with a 750-µm interval according to the following formula: 0.75 * (A_1_+A_2_+A_3_+…+A_n_).

### Iba-1 activity

For immunohistochemical staining for activated microglia, cryosections were fixed in 4% paraformaldehyde in phosphate buffered saline and then incubated with blocking serum at room temperature. Sections were incubated overnight at room temperature with an anti-Iba-1 antibody (rabbit, 1∶1,500, WAKO Pure Chemical Industries, Osaka, Japan). The secondary antibody was a biotinylated anti-rabbit IgG (H+L) BA-1000 (Vector Laboratories Inc., Burlingame, Ca, USA). The total amount of positive cells was counted in the cortical percontusional tissue (ROI: 0.2×0.3 mm) at bregma −1.28 mm according to a mouse brain atlas (www.mbl.org) by an investigator blinded towards the group allocation of the animals (see Figure 2 E).

### Quantification of functional outcome

The before- and after-CCI functional outcome was determined by an investigator blinded towards the experimental groups by measuring the body weight and assessing a neuroscore. The 10-point neurological severity score (NSS) was applied as described by Tsenter et al. [31]. The neuroscore consists of 10 different tasks that evaluate motor ability, alertness, balance, and general behavior. For each failed task, the mouse receives 1 point. Healthy mice were successful in all tasks and received 0 points. Severely impaired animals received up to 10 points (**Table 2**).

### RNA isolation, quality control and cDNA synthesis

RNA was isolated from brain tissue samples, which were collected from the right upper quadrant (contused brain tissue) during the histological processing. Samples were lysed with RNeasy lysis buffer (Qiagen, Hilden, Germany) and homogenized with a MM300 mill mixer (Retsch, Haan, Germany) operated at 30 Hertz for 2 minutes. Total RNA was isolated from the lysed and homogenized cells using the RNeasy Lipid Tissue Mini Kit (Qiagen) according to the manufacturer's instructions and eluted with 25 µL RNase-free water. RNA concentrations were spectrometrically calculated using NanoVue (GE Healthcare Europe, Munich, Germany). Then, 0.5 µg extracted RNA was reverse-transcribed into cDNA using the Verso™ cDNA Kit (ABgene, Hamburg, Germany) according to the manufacturer's instructions.

### PCR standard generation for absolute qPCR quantification

Fragments of all applied genes were generated by PCR on a Thermocycler gradient (Eppendorf, Hamburg, Germany). The PCR cycle parameters were as follows: thermal activation for 10 min at 95°C and 35 cycles of PCR (melting for 45 sec at 94°C, annealing for 45 sec at 55–65°C, and extension for 60 sec at 72°C). The primers used [30], [32] are listed in Table 3. To verify the specificity of the PCR reactions, PCR products were electrophoresed alongside the 50-bp DNA Molecular Weight Marker XIII (Roche Diagnostics, Mannheim, Germany) through a 2% (w/v) agarose gel (Invitrogen). The gels were stained with SYBR green (Roche), and images were captured using a Kodak EDAS 120 Image System (Eastman Kodak Sàrl, Genève, Switzerland). The PCR products were purified with the QIA quick PCR Purification Kit (Qiagen) according to the manufacturer's instructions, and the DNA concentrations were determined using a NanoVue system (GE Healthcare). The copy number was calculated and serial 10-fold dilutions were made in the range of 1×10^1^ to 1×10^7^ copies.

**Table 3:** Specific primer and probes and optimized temperature conditions for real-time PCR.

PCR assay	Oligonucleotide Sequence	GenBank No.
[amplicon size, annealing temp]	(5′-3′)	
IL1β	Forw: 5′-gTgCTgTCggACCCATATgAg-3′	NM_008361
[348 bp, 55°C, A: 10 s, E: 15 s]	Rev: 5′-CAggAAgACAggCTTgTgCTC-3′	
	Red: R610-CAgCTggAgAgTgTggATCCCAAgC-Phos	
	FL: 5′-TAATgAAAgACggCACACCCACCC-FL	
IL6	Forw: 5′-GAGGATACCACTCCCAACAGACC-3′	NM_031168
[141 bp, 58°C, A: 10 s, E: 15 s]	Rev: 5′-AAGTGCATCATCGTTGTTCATACA-3′	[31]
iNOS (NOS2)	Forw: 5′-TGTGTCAGCCCTCAGAGTAC-3′	NM_010927
[312 bp, 58°C, A: 10 s, E: 15 s]	Rev: 5′-CACTgACACTYCgCACAA-3′	[29]
	Red: R610-gCTCCTCCCAggACCACACCC-Phos	
	FL: 5′-gAAgCCCCgCTACTACTCCATC-FL	
Cyclophilin A (PPIA)	Forw: 5′-gCgTCTSCTTCgAgCTgTT-3′	NM_008907
[146 bp, 55°C, A: 10 s, E: 15 s]	Rev: 5′-RAAgTCACCACCCTggCA-3′	[29]
	Red: R610-TTggCTATAAgggTTCCTCCTTTCACAg-Phos	
	FL: 5′-gCTCTgAgCACTggRgAgAAAggA-FL	
COX-2 (ptgs2)	Forw: 5′-TCTTTgCCCAgCACTTC-3′	NM_011198
[183 bp, 55°C, A: 10 s, E: 15 s]	Rev: 5′-CCTCTCCACCRATgACCTgA-3′	
	Red: R610-ggTCCTCgCTTMTgATCTgTCTTgAA-Phos	
	FL: 5′-CCAgTCCTCgggTgAACCC-FL	

Forw = sense primer; Rev = anti-sense primer, R610 = Lightcycler® Red610, Phos = phosphate, FL = fluorescein.

### Quantitative polymerase chain reaction (real-time PCR)

The cDNAs from each sample were amplified by a real-time Lightcycler 480 PCR System (Roche). Equal amounts of cDNA (1 µl) were used in duplicate and amplified with a Lightcycler® 480 Probes Master or Lightcycler® 480 SYBR Green I Master (Roche). Real-time cycle parameters were as follows: thermal activation for 10 min at 95°C and 50 cycles of PCR (melting for 10 sec at 95°C, annealing for 10 sec at 55°C (for HybProbe assays) or, according to Table 3, extension for 15 sec at 72°C). Applied primers and probes are listed in Table 3. A standard curve for absolute quantification was generated with PCR DNA for each PCR product (10^1^–10^7^). The absolute copy numbers of the target genes were normalized against the absolute copy numbers of Cyclophilin A (PPIA) as the control gene [30].

### Statistical Analysis

Histological brain damage, neurological function score, gene expression (IL6, COX-2, iNOS), and microglia activation (Iba-1) were compared between the different anesthetics (iso vs. sevo, iso vs. comb, and sevo vs. comb) with Wilcoxon Mann Whitney Rank Sum tests, and p-values were adjusted for multiple comparisons (Bonferroni adjustment). Physiologic data of each time point were analyzed by one-way ANOVA. Results are presented as mean ± standard deviation (S.D.). If not indicated otherwise, adjusted p-values were considered significant at the p<0.05 level. However, as the study was conducted in an explorative approach, all p-values were considered as explorative.

Statistical analysis was performed with the SigmaStat 3.11 Statistical Software package (SPSS Science, USA).
